# The Ax21 protein influences virulence and biofilm formation in *Stenotrophomonas maltophilia*

**DOI:** 10.1007/s00203-017-1433-7

**Published:** 2017-09-30

**Authors:** Shi-qi An, Ji-liang Tang

**Affiliations:** 1 Division of Molecular Microbiology, School of Life Sciences, University of Dundee, Dundee, UK; 2 College of Life Science and Technology, Guangxi University, 100 Daxue Road, Nanning, Guangxi 530004 China

**Keywords:** *Stenotrophomonas*, Antibiotic resistance, Motility, Biofilm formation, Virulence

## Abstract

*Stenotrophomonas maltophilia* is an antibiotic-resistant Gram-negative pathogen, which is associated with hospital-acquired infection. The genome encodes a protein highly related to the Ax21 protein of *Xanthomonas oryzae* that is implicated in interactions of this plant pathogen with rice. Here, we report on the pleiotropic nature of *ax21* mutation in *S. maltophilia* and the effects of addition of the Ax21 protein on the restoration of the wild-type phenotype. We show that loss by mutation of Ax21 leads to reduced motility, reduced biofilm formation, reduced tolerance to the antibiotic tobramycin and reduced virulence to larvae of *Galleria mellonella*, as well as alteration in the expression of specific genes associated with virulence or antibiotic resistance. Addition of the Ax21protein restored motility and the level of gene expression towards wild type. These findings are consistent with the notion that the Ax21 protein is involved in intraspecies communication, although other interpretations cannot be discounted.

## Introduction

Ax21 is an outer membrane protein that is extensively conserved in plant pathogenic *Xanthomonas* and the associated genera *Xylella* and *Stenotrophomonas*, including *S. maltophilia*, some strains of which are hospital-acquired human pathogens (Ryan et al. 2009). Studies on Ax21 in *Xanthomonas oryzae* pv. *oryzae* (*Xoo*) have shown that the protein can be sulphated and that it is secreted into the bacterial medium in association with the outer membrane vesicles (Han et al. 2012; Bahar et al. 2014). Although a sulphated peptide derived from the N-terminus of Ax21 was originally thought to be a specific trigger for XA21-dependent innate immunity in rice, subsequent work has shown that this is not the case. Nevertheless, this peptide does induce defence-related responses in plants (Danna et al. 2011). A second proposed role for Ax21 is as a diffusible signal that controls the gene expression in *Xoo* as a response to bacterial cell density (Bahar et al. 2014). These observations led us to examine the potential role(s) of Ax21 in *S. maltophilia.* In 2011, it was reported that the mutation of *ax21* had effects on different phenotypes in *S. maltophilia* (McCarthy et al. 2011). However, this paper was recently retracted due to errors in data presentation of Fig. 2 (McCarthy et al. 2017). Here, we report on the outcomes of repeated key experiments that indicate the pleiotropic nature of *ax21* mutation and the effects of addition of the Ax21 protein on the restoration of the wild-type phenotype. Here, our aim was to repeat key experiments that indicate the pleiotropic nature of *ax21* mutation and the effects of the addition of the Ax21 protein on the restoration of the wild-type phenotype.

## Materials and methods

### Bacterial strains and growth conditions

The wild-type *S. maltophilia* was strain K279a (Crossman et al. 2008). Unmarked deletion of *smlt0387* (designed as *ax21*) was constructed in *S. maltophilia* K279a strain using the pEX18Gm suicide vector, which uses the *sacB*-based counterselection method (Hoang et al. 1998). To delete *ax21*, the following procedure was used: a PCR fragment containing 150 nucleotides upstream of the *ax21* start site and 150 nucleotides downstream were amplified and then inserted into pEX18Gm that had been digested with the same enzymes and linked to a *BamHI*–*HindIII*-digested PCR fragment. The pEX18Gm–ax21 construct was sequenced to confirm that PCR did not introduce a mutation before the construct was mobilized from *E. coli* into *S. maltophilia* K279a. The transconjugants carrying the integrated plasmid on the chromosome were selected on l-agar plates containing 10% (wt/vol) sucrose (Oxoid, UK) and 25 μg/mL gentamicin (Fisher, UK). The resistant colonies were screened using colony PCR to identify mutants. For complementation studies, the *smlt0387* gene was cloned into pBBR1MCS (Kovach et al. 1995). Strains and plasmids used during this study are detailed in Table 1. For the majority of the experiments, the NYGB medium was used as growth medium for *S. maltophilia* strains, which comprises 20 g/L glycerol (Oxoid, UK), 3 g/L yeast extract (Difco, UK) and 5 g/L bacteriological peptone (Oxoid, UK). The assessment of bacterial clumping or biofilm formation was carried out in L medium, which comprises sodium chloride, 5 g/L; yeast extract, 5 g/L; Bactotryptone (Difco, UK), 10 g/L and d-glucose (Fisher, UK), 1 g/L. Peptides Ax21 (Smlt0387) and Ax21Y (Smlt0387 with Y altered to A) used in the experiments were generated by Cambridge Peptides (http://www.cambridgepeptides.com) and at 500 nM unless otherwise stated.

**Table 1:** Bacterial strains and plasmids used in this work

Strain or plasmid	Relevant characteristics	Source or references
*Stenotrophomonas maltophilia*		
K279a	Clinical isolate	Crossman et al. (2008)
K279a *ax21*	*smlt0387* mutant of K279a	This study
ax21(pSmlt0387)	ax21 mutant complemented with smlt0387 using pBBR1MCS	This study
Plasmids		
pEX18Gm	Broad-host-rang allelic exchange vector, Gm^r^	Hoang et al. (1998)
pBBR1MCS	Broad-host-range cloning vector, Cm^r^	Kovach et al. (1995)

### RNA extraction and qRT-PCR

For RNA extractions, *S. maltophilia* strains were cultivated at 30 °C in NYGB broth (without antibiotic) to logarithmic phase (OD_600_ ≈ 0.8). A volume of 800 μL of RNA protect (Qiagen, UK) was added to 400 μL of culture and incubated at room temperature for 5 min. These suspensions were centrifuged and the resulting pellets were stored at −80 °C after removal of the supernatant. Following the manufacturer’s instructions, total RNA was isolated from cells after thawing, using the RNeasy Mini Kit (Qiagen, UK) and then treated with DNase (Ambion, UK). PCR was used to confirm the removal of DNA contamination. Specific RT-PCR primers were used to amplify the central fragments of approximately 200 bp in length from *smlt1112* (Frd-AGGACCCCTGGAACGTTTG; Rev-CACATCCGGCACCACATAGG), *smlt1390* (Frd-AGTTGGGCATCAACACCGAT; Rev-GGGTTGCCTTCTTGCTCTGA), *smlt2175* (Frd-AGCCAGAAGGAAACCACCTG; Rev-GCGGTCATAGGTCTGCACTT) and *smlt3949* (Frd-TTCCAGTTCGATAACGCCGC; Rev-CTCAGGCGACCCACATACAA). Quantification of gene expression was assessed using a Rotor-Gene Q (Qiagen, UK) and QuantiFast SYBR Green PCR Kit (Qiagen, UK). Furthermore, the expression of genes encoding the RNA polymerase sigma factor RpoN (Smlt1112), a putative outer membrane surface haemagglutinin (Smlt1390), putative TonB receptor (Smlt2175) and putative two-component regulator TctD (Smlt3949) were analysed because of their known role in virulence and antibiotic resistance in *Stenotrophomonas* (Devos et al. 2015; Ferrer-Navarro et al. 2016).

### Motility assays

Bacterial motility assays were carried out on NYGB medium that was solidified using 0.6% Eiken agar (Eiken Chemical, Tokyo). A sterile 200-µl tip was used to inoculate *S. maltophilia* strains to the centre of the plate. The plates were visualized after incubation at 30 °C for 48 h.

### Biofilm formation assay

Biofilm development was assessed on glass by crystal violet staining as described in O’Toole and Kolter (1998). *Stenotrophomonas maltophilia* strains were cultivated to logarithmic phase and then diluted to an OD at 600 nm of 0.1 in L medium. A volume of 5 mL of culture was incubated at 30 °C for 24 h in static glass tubes (14 mL). Once the medium and unattached cells were removed, adherent bacteria were washed twice with sterile water and then stained with 0.1% (w/v) crystal violet (Fisher, UK). Water was used to remove all unbound dye. The bound crystal violet was quantified by solubilizing in ethanol and reading at 595 nm.

### Antibiotic killing curves

Killing curves were carried out at 30 °C as previously described by Macfarlane et al. (1999). *Stenotrophomonas maltophilia* strains were grown to mid-log phase on NYGB and then diluted to 10^6^ in 100 mL of pre-warmed PBS containing the aminoglycoside tobramycin (Sigma, UK) at 100 μg/mL. Similarly, an antibiotic-free control was inoculated. At 0, 10, 20, 30, 50, 100, 120 and 180 min after antibiotic exposure, 0.1 mL volumes were removed, diluted in PBS and inoculated onto NYG agar plates. To determine viable CFU, these plates were incubated for 24 h at 30 °C.

### Virulence assay

*Galleria mellonella* larvae were stored at 4 °C in wood shavings. For experiments, live versus dead larvae were observed after 24 h post-infection. *Galleria mellonella* were injected with 10 μL of successively diluted bacteria (1 × 10^6^ CFU). Infected *G. mellonella* were placed on Whatman paper-lined Petri dishes and incubated at 37 °C. The *G. mellonella* were monitored for their survival after a 24-h period. Three separate tests were conducted consisting of ten larvae for each strain. The control groups for each experiment consisted of *G. mellonella* injected with PBS alone and a group of uninfected *G. mellonella*.

## Results and discussion

The predicted proteome of *S. maltophilia* K279a, a clinical isolate (Crossman et al. 2008), contains two proteins that are homologous to Ax21 of *Xoo* strain PXO99^A^: Smlt0387 (BLASTP probability score e-77) and Smlt0184 (e-62). Interestingly, Ax21 homologue Smlt0387 was recently identified in *S. maltophilia* to be secreted in association with outer membrane vesicles, which play roles in both infection and antibiotic resistance (Devos et al. 2015; Ferrer-Navarro et al. 2016).

**Fig. 1:**
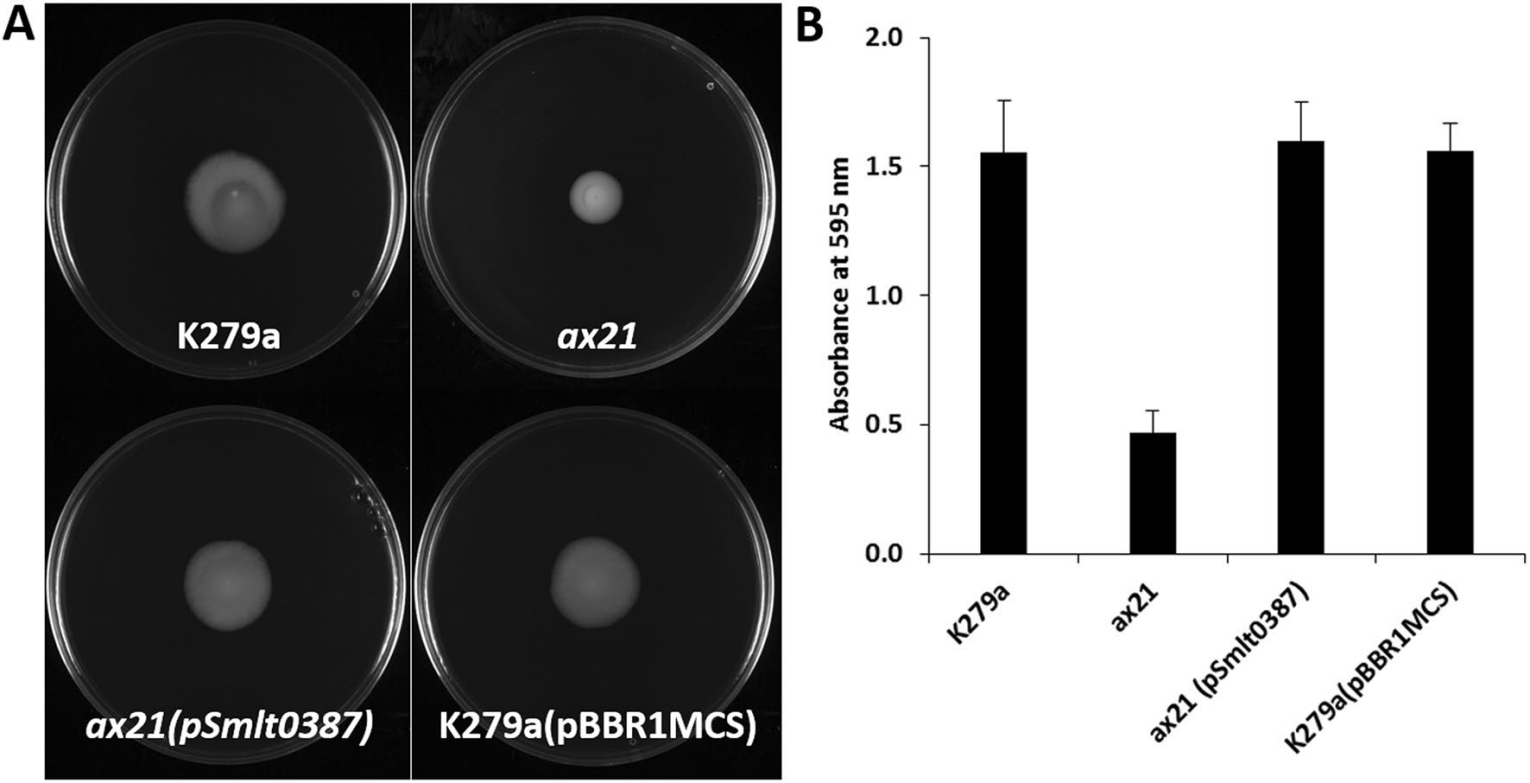
Mutation of *ax21* has pleiotropic effects in *S. maltophilia* K279a. **a** The *ax21* mutant shows reduced motility in 0.6% Eiken agar, and complementation with *smlt0387* in *trans* restores motility to wild type. **b** The *ax21* mutant shows reduced biofilm formation on glass as quantified by crystal violet staining. Complementation with *smlt0387* in *trans* restores motility to wild type

**Fig. 2:**
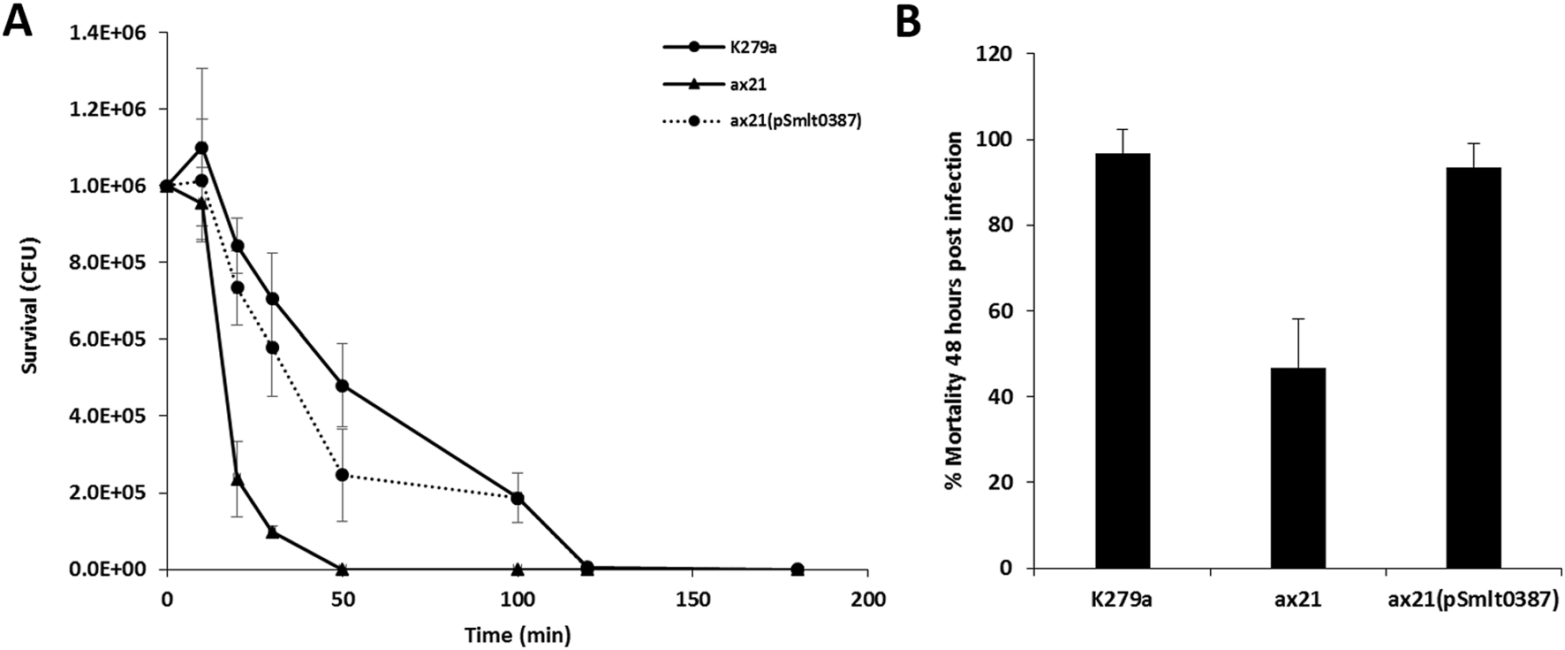
Mutation of *ax21* has effects on antibiotic tolerance and virulence in *S. maltophilia* K279a. **a** The *ax21* mutant shows reduced tolerance to the aminoglycoside tobramycin at 100 μg/mL as revealed by a killing curve. **b** The *ax21* mutant shows reduced virulence in the *G. mellonella* larva infection model. These mutant phenotypes could be restored to wild-type levels in all cases through complementation by *in trans* expression of a wild-type copy of the gene

The possible role of Ax21 in *S. maltophilia* was initially assessed by examination of the effect of deletion of *smlt0387* on a number of phenotypes. Deletion of *smlt0387* had a pleiotropic effect, leading to reduced motility on 0.6% Eiken agar (Fig. 1a), reduced biofilm formation on a glass surface (Fig. 1b), reduced tolerance to the aminoglycoside antibiotic tobramycin (Fig. 2a) and reduced virulence to larvae of *Galleria mellonella* (Fig. 2b). *In trans* complementation restored these altered phenotypes to the wild-type phenotype (Figs. 1 and 2). The pleiotropic effects of loss by mutation Ax21 are consistent with previous observations in different pathovars of *Xanthomonas oryzae*, where *ax21* mutants have altered biofilm formation, extracellular polysaccharide synthesis and virulence (Qian et al. 2013; Park et al. 2014). Furthermore, a correlation has been shown between the abundance of Ax21 in different strains of *S. maltophilia* and the mortality rate when those strains were tested in a Zebrafish model of infection (Ferrer-Navarro et al. 2013, 2016). Curiously, analysis of a strain with mutation in *smlt0184* did not show any alteration in the phenotypes tested.

**Fig. 3:**
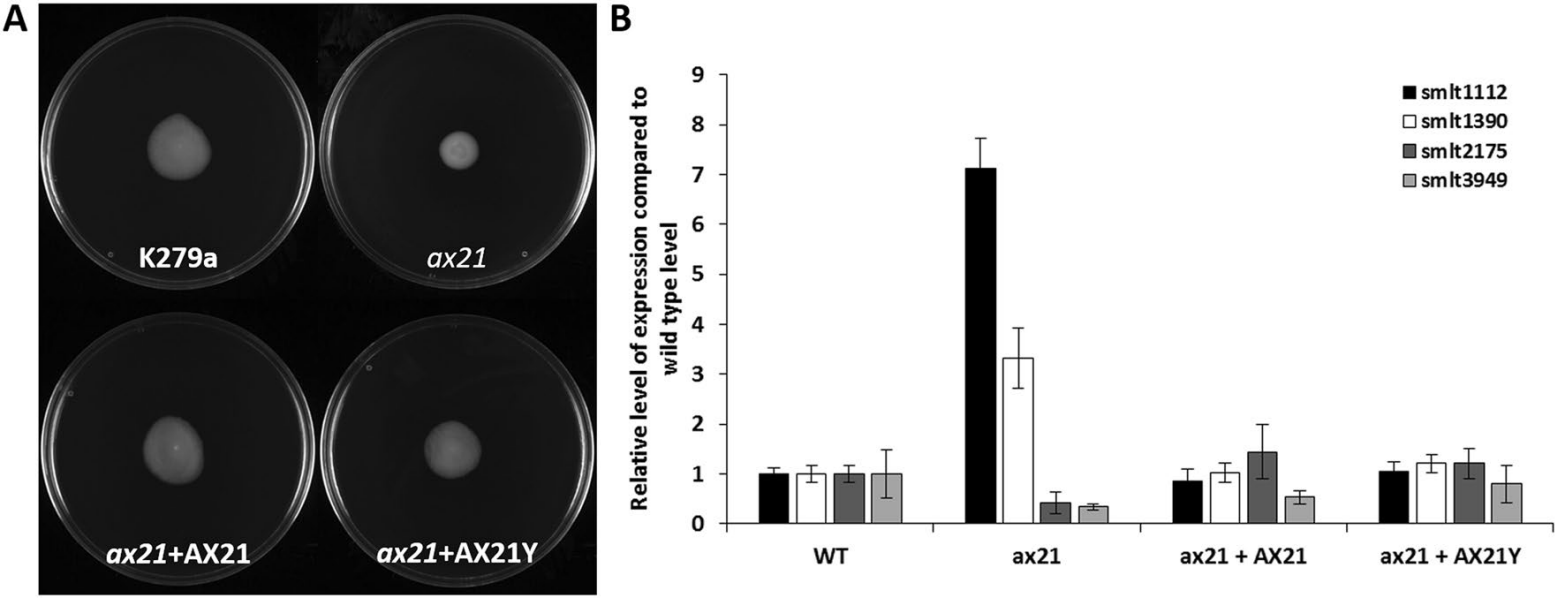
Exogenous Ax21 and the non-sulphatable Y22A variant form of Ax21 (here designated AX21Y) are active in the regulation in *S. maltophilia*. **a** Exogenous addition of either AX21 or AX21Y to the medium restored motility to an *ax21* mutant. **b** Exogenous addition of either AX21 or AX21Y to the *ax21* mutant restored the level of expression of *smlt1112, smlt1390, smlt2175 and smlt3949* towards wild type as measured by qRT-PCR

For a number of bacterial cell-to-cell signalling systems, the phenotypic effects caused by deletion of the gene encoding the signal synthetase can be reversed by exogenous addition of the signal molecule (Papenfort and Bassler 2016). To test this potential role for Ax21, we repeated the motility tests in the presence of a synthetic Ax21 protein. Addition of the protein at 500 nM restored wild-type motility (Fig. 3a). A variant Ax21 protein (Ax21Y) in which the tyrosine residue that is sulphated in Ax21 of *Xoo* was replaced by an alanine residue also restored motility (Fig. 3a). This is intriguing, since *S. maltophilia* lacks homologues of RaxST believed to be responsible for the sulphation of Ax21 in *Xoo*.

Quantitative reverse transcription polymerase chain reaction (qRT-PCR) was then used to establish if the phenotypic effects of *smlt0387* deletion were associated with specific changes in gene expression. The findings (Fig. 3b) showed that loss of *ax21* led to elevated expression of Smlt1112 and Smlt1390, but decrease in the expression of Smlt2175 and Smlt3949. Addition of Ax21 or Ax21Y restored the expression of all of these genes towards wild type.

The effects of Ax21 on *S. maltophilia* thus appear to extend beyond changes that may influence the production or degradation of the molecule, consistent with the notion that the Ax21 protein is a signal involved in intraspecies communication (Winzer et al. 2002). However, other interpretations of the findings cannot be discounted. For example, pleiotropic effects may occur if loss of Ax21 causes dysfunction of the outer membrane leading to cell stress. Furthermore, work in *Xanthomonas* and *Stenotrophomonas* has indicated an influence of the DSF (Diffusible Signal Factor) cell-to-cell signal on the synthesis or secretion of Ax21 (Qian et al. 2013; Devos et al. 2015), raising the possibility that Ax21 acts indirectly through an influence on DSF signalling.

## Conclusions

Ax21 influences a diverse range of functions in the nosocomial pathogen *S. maltophilia* leading to altered virulence, tobramycin tolerance and biofilm formation. Further work is needed to establish whether Ax21 is truly a cell–cell signal.
